# Value and reward based learning in neurorobots

**DOI:** 10.3389/fnbot.2013.00013

**Published:** 2013-09-13

**Authors:** Jeffrey L. Krichmar, Florian Röhrbein

**Affiliations:** ^1^Department of Cognitive Sciences, Department of Computer Science, University of CaliforniaIrvine, CA, USA; ^2^Department of Informatics VI, Technische Universität MünchenGarching, Germany

**Keywords:** value system, neuromodulation, reinforcement learning, action selection, neurorobotics, reward-based learning, basal ganglia, embodied cognition

Organisms are equipped with value systems that signal the salience of environmental cues to their nervous system, causing a change in the nervous system that results in modification of their behavior. These systems are necessary for an organism to adapt its behavior when an important environmental event occurs. A value system constitutes a basic assumption of what is good and bad for an agent. These value systems have been effectively used in robotic systems to shape behavior. For example, many robots have used models of the dopaminergic system to reinforce behavior that leads to rewards. Other modulatory systems that shape behavior are acetylcholine's effect on attention, norepinephrine's effect on vigilance, and serotonin's effect on impulsiveness, mood, and risk. Moreover, hormonal systems such as oxytocin and its effect on trust constitute as a value system. A recent Research Topic in Frontiers of Neurorobotics explored value and reward based learning. The topic comprised of nine papers on research involving neurobiologically inspired robots whose behavior was shaped by value and reward learning, adapted through interaction with the environment, or shaped by extracting value from the environment.

Value systems are often linked to reward systems in neurobiology and in modeling. For example, Jayet Bray and her colleagues developed a neurorobotic system that learned to categorize the valence of speech through positive verbal encouragement, much like a baby would (Jayet Bray et al., 2013). Their virtual robot, which interacted with a human partner, was controlled by a large-scale spiking neuron model of the visual cortex, premotor cortex, and reward system. An important issue in both biological and artificial reward systems is the credit assignment problem that is, how can a distal cue be linked to a reward. In other words, how can you extract the stimulus that predicts a future reward from all the noisy stimuli that you are faced with? Soltoggio and colleagues introduce the principle of rare correlations to resolve this issue (Soltosggio et al., 2013). By using Rarely Correlating Hebbian Plasticity, they demonstrated classical and operant conditioning in a set of human-robot experiments with the iCub robot.

The notion of value and reward has often been formalized in reinforcement learning systems. For example, Li and colleagues show that reinforcement learning, in the form of a dynamic actor-critic model, can be used to tune central pattern generators in a humanoid robot (Li et al., 2013). Through interaction with the environment, this dynamical system developed biped locomotion on a NAO robot that could adapt its gaits to different conditions. Elfwing and colleagues introduced a scaled version of free-energy reinforcement learning (FERL) and applied it to visual recognition and navigation tasks (Elfwing et al., 2013). This novel algorithm was shown to be significantly better than standard FERL and feedforward neural network RL. Another related method, Linearly solvable Markov Decision Process (LMDP) has been shown to have advantages over RL in optimal control policy (Kinjo et al., 2013). Kinjo and colleagues demonstrated the power of LMDP for robot control by applying the method to a pole balancing task, and a visually guided navigation problem using their Spring Dog robot which has six degrees-of-freedom.

Value does need not be reward-based; curiosity, harm, novelty, and uncertainty can all carry a value signal. For example, in a biomimetic model of the cortex, basal ganglia and phasic dopamine, Bolado-Gomez and colleagues (Bolado-Gomez and Gurney, 2013) showed that intrinsically motivated operant learning (i.e., action discovery) could replicate rodent experiments, in a virtual robot. In this case, phasic dopaminergic neuromodulation carried a novelty salience signal, rather than the more conventional reward signal. In a model called CURIOUSity-DRiven, Modular, Incremental Slow Feature Analysis (Curious Dr. MISFA), Luciw and colleagues showed that curiosity could shape the behavior of an iCub robot in a multi-context environment (Luciw et al., 2013). Their model was inspired by cortical regions of the brain involved in unsupervised learning, as well as neuromodulatory systems responsible for providing intrinsic rewards through dopamine and regulating levels of attention through norepinephrine. Different neuromodulatory systems in the brain may be related to different aspects of value (Krichmar, 2013). In a model of multiple neuromodulatory systems, Krichmar showed that interactions between the dopaminergic (reward), serotoninergic (harm aversion), and the cholinergic/noradrenergic (novelty) systems could lead to interesting behavioral control in an autonomous robot. Finally, in an interesting position paper, Friston, Adams, and Montague suggest that *value is evidence*, specifically log Bayesian evidence (Friston et al., 2012). They propose that reward or cost functions that underlie value in conventional models of optimal control can be cast as prior beliefs about future states, which is simply accumulation of evidence through Bayesian updating of posterior beliefs.

As can be gleaned from reading the papers in the Research Topic, as well as the empirical evidence and studies they are built on, Value and Reward Based Learning is an active and broad area of research. The application to neurorobotics is important for several reasons: (1) It provides an embodied platform for testing hypotheses regarding the neural correlates of value and reward, (2) it provides a means to test more theoretical hypotheses on the acquisition of value and its function for biological and artificial systems, and (3) it may lead to the development of improved learning systems in robots and other autonomous agents.

## References

[B1] Bolado-Gomez R. Gurney K. (2013). A biologically plausible embodied model of action discovery. Front. Neurorobot. 7:4 10.3389/fnbot.2013.00004 23487577 PMC3594743

[B2] Elfwing S. Uchibe E. Doya K. (2013). Scaled free-energy based reinforcement learning for robust and efficient learning in high-dimensional state spaces. Front. Neurorobot. 7:3 10.3389/fnbot.2013.00003 23450126 PMC3584292

[B3] Friston K. Adams R. Montague R. (2012). What is value-accumulated reward or evidence? Front. Neurorobot. 6:11 10.3389/fnbot.2012.00011 23133414 PMC3487150

[B4] Jayet Bray L. C. Ferneyhough G. B. Barker E. R. Thibeault C. M. Harris F. C. Jr (2013). Reward-based learning for virtual neurorobotics through emotional speech processing. Front. Neurorobot. 7:8 10.3389/fnbot.2013.00008 23641213 PMC3638126

[B5] Kinjo K. Uchibe E. Doya K. (2013). Evaluation of linearly solvable Markov decision process with dynamic model learning in a mobile robot navigation task. Front. Neurorobot. 7:7 10.3389/fnbot.2013.00007 23576983 PMC3617398

[B6] Krichmar J. L. (2013). A neurorobotic platform to test the influence of neuromodulatory signaling on anxious, and curious behavior. Front. Neurorobot. 7:1 10.3389/fnbot.2013.00001 23386829 PMC3564231

[B7] Li C. Lowe R. Ziemke T. (2013). Humanoids learning to walk:a natural CPG-actor-critic architecture. Front. Neurorobot. 7:5 10.3389/fnbot.2013.00005 23675345 PMC3619089

[B8] Luciw M. Kompella V. Kazerounian S. Schmidhuber J. (2013). An intrinsic value system for developing multiple invariant representations with incremental slowness learning. Front. Neurorobot. 7:9 10.3389/fnbot.2013.00009 23755011 PMC3667249

[B9] Soltosggio A. Lemme A. Reinhart F. Steil J. J. (2013). Rare neural correlations implement robotic conditioning with delayed rewards and disturbances. Front. Neurorobot. 7:6 10.3389/fnbot.2013.00006 23565092 PMC3613617

